# Assessment and rehabilitation of central sensory impairments for balance in mTBI using auditory biofeedback: a randomized clinical trial

**DOI:** 10.1186/s12883-017-0812-7

**Published:** 2017-02-23

**Authors:** Peter C. Fino, Robert J. Peterka, Timothy E. Hullar, Chad Murchison, Fay B. Horak, James C. Chesnutt, Laurie A. King

**Affiliations:** 1 Department of Neurology, Oregon Health & Science University, 3181 SW Sam Jackson Park Road, L226, Portland, OR 97239-3098 USA; 2 Department of Biomedical Engineering, Oregon Health & Science University, Portland, Oregon USA; 3 Department of Otolaryngology – Head and Neck Surgery, Oregon Health & Science University, Portland, Oregon USA; 4 Department of Orthopaedics and Rehabilitation, Oregon Health & Science University, Portland, Oregon USA; 5 National Center for Rehabilitative Auditory Research, Veterans Affairs Portland Health Care System, Portland, Oregon USA; 6 Veterans Affairs Portland Health Care System, Portland, Oregon USA

**Keywords:** Sensorimotor integration, mTBI, Concussion, Balance, Gait, Biofeedback

## Abstract

**Background:**

Complaints of imbalance are common non-resolving signs in individuals with post-concussive syndrome. Yet, there is no consensus rehabilitation for non-resolving balance complaints following mild traumatic brain injury (mTBI). The heterogeneity of balance deficits and varied rates of recovery suggest varied etiologies and a need for interventions that address the underlying causes of poor balance function. Our central hypothesis is that most chronic balance deficits after mTBI result from impairments in central sensorimotor integration that may be helped by rehabilitation. Two studies are described to 1) characterize balance deficits in people with mTBI who have chronic, non-resolving balance deficits compared to healthy control subjects, and 2) determine the efficacy of an augmented vestibular rehabilitation program using auditory biofeedback to improve central sensorimotor integration, static and dynamic balance, and functional activity in patients with chronic mTBI.

**Methods:**

Two studies are described. Study 1 is a cross-sectional study to take place jointly at Oregon Health and Science University and the VA Portland Health Care System. The study participants will be individuals with non-resolving complaints of balance following mTBI and age- and gender-matched controls who meet all inclusion criteria. The primary outcome will be measures of central sensorimotor integration derived from a novel central sensorimotor integration test. Study 2 is a randomized controlled intervention to take place at Oregon Health & Science University. In this study, participants from Study 1 with mTBI and abnormal central sensorimotor integration will be randomized into two rehabilitation interventions. The interventions will be 6 weeks of vestibular rehabilitation 1) with or 2) without the use of an auditory biofeedback device. The primary outcome measure is the daily activity of the participants measured using an inertial sensor.

**Discussion:**

The results of these two studies will improve our understanding of the nature of balance deficits in people with mTBI by providing quantitative metrics of central sensorimotor integration, balance, and vestibular and ocular motor function. Study 2 will examine the potential for augmented rehabilitation interventions to improve central sensorimotor integration.

**Trial registration:**

This trial is registered at clinicaltrials.gov (NCT02748109)

## Background

Between 1.6 and 3.8 million sports-related mild traumatic brain injuries (mTBI) occur annually in the United States [1] and up to 15% of military combat veterans sustain mTBI [2]. The estimated annual cost of TBI, including lost productivity, exceeds $60 billion [3]. While self-reported symptoms of mTBI typically recover within three months [4], between 11 and 64% of individuals, depending on diagnostic criteria, develop post-concussive syndrome with persistent, non-resolving symptoms [5]. Despite the high incidence and cost to society, studies investigating the treatment of chronic mTBI symptoms have been scarce. In particular, complaints of imbalance are common following mTBI [6, 7] and are significant contributors to anxiety and difficulty returning to work [8]. Yet, there is currently no consensus, evidence-based rehabilitation procedure for balance complaints following mTBI [9].

**Fig. 1:**
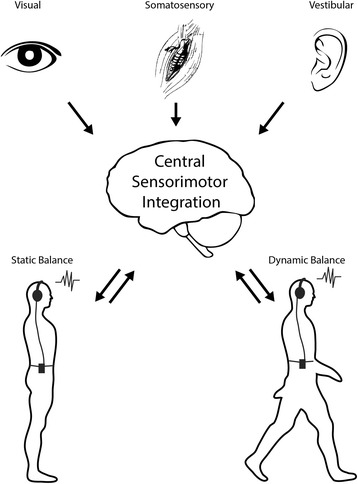
Sensory systems contributing to static and dynamic balance

The difficulty establishing consensus rehabilitation protocols is likely due to the complex nature of balance deficits in individuals with mTBI that can include neurological damage to the brainstem [10, 11], thalamus [12], and cerebellum [13], and / or injury to peripheral vestibular organs [14–17]. Sensory information from the vestibular, visual, and somatosensory systems are integrated in the central nervous system to control static and dynamic balance (Fig. 1). In patients with mTBI, vestibular function can be impaired by damage to the peripheral vestibular organs. The semicircular canals can be impaired in up to two-thirds of individuals with mTBI [15, 17], and the otolith organs are also susceptible to damage from mTBI [14, 16]. In addition to disrupting vestibular function, mTBI has also been linked with central deficits affecting ocular motor function including extended saccadic latency [18] and poor visual tracking accuracy during smooth pursuit tasks [19, 20]. In one study, 60% of individuals with mTBI had errors during pursuit whereas no controls exhibited similar errors [21].

A proportion of individuals with mTBI who do not have prominent or measurable sensory deficits may still have balance problems attributable to abnormal central control of balance. Central sensorimotor integration is a critical aspect of balance control that may underlie dysfunction in static and dynamic balance. Proper central sensorimotor integration relies on the central nervous system to 1) select the optimal combination of sensory sources for balance and 2) reweight the sensory contributions as sensory conditions change [22, 23]. When peripheral vestibular, visual, and somatosensory systems are intact, difficulty performing static or dynamic balance tasks under challenging surface and/or visual conditions may be due to centrally-mediated suboptimal weighting and/or reweighting of vestibular, visual, and somatosensory information or due to abnormal sensory-to-motor transformations that inappropriately scale the magnitude of corrective motor actions [22–24]. For example, standing or walking on compliant foam or irregular surfaces requires the nervous system to rely more on vestibular and visual inputs while down-weighting the normal dependence on somatosensory information [22]. This reweighting is also important for individuals with sensory impairment, as altered patterns of weighting are necessary to compensate for the sensory dysfunction. Some patterns of compensation may be more functional than others, suggesting an important role of rehabilitation in helping subjects develop effective compensation strategies to improve recovery of central sensorimotor integration and balance [24]. However, central sensorimotor integration has not been systematically characterized in patients with mTBI, regardless of sensory loss.

The lack of knowledge about central sensorimotor integration following mTBI may be in part due to the current clinical tools used to assess balance, gait, and sensorimotor integration. The Balance Error Scoring System (BESS) is the most frequently administered clinical balance assessment for patients with suspected mTBI. Yet, the BESS suffers from a high degree of subjectivity, provides only limited, low-resolution information about the balance control system [25], and is not sensitive beyond the acute stages of mTBI [26]. Balance measures using force platforms and sophisticated post-processing and analysis techniques have shown greater sensitivity in detecting long-lasting postural deficits [27–31]. Similar to static balance, measures of dynamic balance, such as trunk motion during gait [32–35] have shown persistent impairment post-concussion. Sensitive, objective measures of balance can be obtained using wearable sensors [36–39] and are well-suited for clinical and rehabilitation environments [40, 41].

For instance, wearable sensors enable biofeedback based on quantitative measurements of balance and gait. Specifically, auditory biofeedback (ABF), which communicates sway through changes in the pitch or location of a tone, has shown promise in aiding balance and retraining central sensory integration mechanisms in populations with balance impairments [42, 43]. Quantitative measures of sway and body position are communicated to the patient via audition, in real-time, to augment existing sensory information and provide accurate information to the central nervous system about body sway that may help recalibrate distorted sensory integration. Compared to visual biofeedback paradigms, ABF is particularly well suited for populations with mTBI as the auditory cue does not interfere with other postural sensory systems, such as vision.

The heterogeneity of balance deficits and varied rates of recovery after mTBI suggest varied etiologies and a need for interventions that address the underlying causes of poor balance function. Over-simplified clinical balance measures often do not account for the complex integration of sensory and motor systems. Therefore, the first goal of this study is to characterize balance deficits in people with mTBI who have chronic, non-resolving balance deficits compared to healthy control subjects without a history of mTBI. We hypothesize that a) objective measures of central sensorimotor integration, static and dynamic balance will better distinguish people with mTBI from control subjects than clinical measures, b) a subset of people with mTBI will have abnormal central sensorimotor integration test measures, even without peripheral vestibular or ocular motor deficits, and c) the relationship between poorer static/dynamic balance performance and mTBI is regulated/mediated by central sensorimotor integration. The second goal is to determine if augmenting a traditional balance rehabilitation program with a wearable-sensor based ABF system improves central sensory integration in patients with chronic mTBI compared to the standard balance rehabilitation program. Our central hypothesis is that chronic balance deficits after mTBI result from impairments in central sensorimotor integration that may be helped by rehabilitation that challenges balance while simultaneously providing feedback on balance performance. We hypothesize that a) central sensorimotor integration scores will improve with rehabilitation and ABF will increase the improvement of central sensorimotor integration scores beyond the standard of care, b) intervention with ABF will improve objective measures of balance and c) people with central sensorimotor integration impairment will show sustained improvement in central sensorimotor integration scores and balance after rehabilitation with ABF.

## Methods / Design

This study has two parts: Study 1) A cross-sectional study to identify and characterize balance control strategies after mTBI compared to healthy controls, and Study 2) An interventional randomized pilot study using a novel ABF rehabilitation technique to improve central sensorimotor integration after mTBI. All subjects will complete baseline clinical sensory, ocular motor, vestibular, neurocognitive, static and dynamic balance, and central sensorimotor integration testing. Following baseline testing, a subset of mTBI participants will be randomized into two treatment groups for 6 weeks of rehabilitation. Within 1 week after completing the rehabilitation program, subjects will repeat the balance and central sensorimotor integration testing to examine the short-term effects of rehabilitation. Subjects will complete the balance and central sensorimotor integration testing a third time, 6 weeks after completion of the rehabilitation, to assess retention. This trial is registered at clinicaltrials.gov (NCT02748109).

### Participants

The study will include 65 mTBI participants and 65 control subjects for the cross-sectional arm of the study. All subjects will be between the ages of 18 and 60. Participants in the mTBI group will have a diagnosed mTBI or concussion and chronic, non-resolving complaints of balance problems determined by a score >0 on the Dizziness Handicap Inventory (DHI) at the time of testing and > 3 months post-injury. Control subjects will be gender and age matched to the mTBI group. For the second arm of the study, a subset of 40 mTBI subjects with abnormal central sensorimotor integration with or without peripheral vestibular or ocular motor deficits will be randomized into two rehabilitation groups, experimental treatment and standard of care, consisting of two rehabilitation sessions per week for 6 weeks. All subjects will be tested at either Oregon Health & Science University or the VA Portland Health Care System. All rehabilitation will take place at Oregon Health & Science University. Informed written consent will be obtained from all participants.

### Inclusion and exclusion criteria

Inclusion criteria for mTBI recruitment are 1) a diagnosis of mTBI based upon VHA/DoD criteria with persisting symptoms >3 months post injury [44], 2) between 21–50 years old, 3) a score >0 on the DHI, 4) minimal cognitive impairment; a score between 0 and 8 on the Short Blessed test for cognitive function [45], and 5) may or may not have had a loss of consciousness (LOC) with their initial injury. Inclusion criteria for controls are between 21–50 years old and no history of mTBI or brain injury. Exclusion criteria are: 1) have had or currently have any other injury, medical, substance or neurological illness that could potentially explain balance deficits (e.g., CNS disease, stroke, moderate TBI, lower extremity amputation) 2) meet criteria for moderate to severe substance use disorder within the past month, as defined by DSM-V, 3) display behavior that would significantly interfere with validity of data collection or safety during study, 4) be in significant pain during the evaluation (5/10 by patient subjective report), 5) be a pregnant female (balance considerations), 6) have past history of peripheral vestibular pathology or ocular motor deficits, 7) have significant hearing loss that would interfere with Study 1 no worse than 60 dB HL (PTA 0.5–3 kHz) and Study 2; hearing loss no worse than 30 dB HL (PTA 0.5–3 kHz), in better ear, with the difference in ears being less than 15 dB PTA, or 8) be unable to abstain for 24 h in advance of testing in the use medications that might impair their balance.

### Study 1: Characterization of balance deficits

#### Assessment procedures

All people who are eligible per phone screening will come into the clinic for the informed consent process. An investigator will verbally explain the consent form, allow the person ample time to read through the consent form and then will acknowledge consent by signing the form. All subjects will first read and sign consent forms. For study 1, 65 mTBI participants and 65 control subjects will be assessed on a battery of tests designed to measure vestibular function, sensorimotor integration, static and dynamic balance, cognition, reaction time, clinical sensory loss, and self-reported symptoms. All protocols have been approved by the OHSU Institutional Review Board.

Participants will complete a novel central sensorimotor integration test (CSMI test) [22] on a modified research NeuroCom platform using custom-designed, low-amplitude (2° and 4° peak-to-peak) pseudorandom stimuli that apply continuously repeated cycles of wide bandwidth surface-tilt and/or visual-tilt stimuli with individual tests lasting ~5 min. The participants’ anteroposterior (AP) body displacement at shoulder and hip levels, and surface and visual surround rotation angles will be recorded. Subjects will perform 8 trials (2 amplitudes x 4 test conditions) in one session with trials presented in a randomized order and interspersed with rest breaks (Table 1). Anthropometric measures will be obtained for the purpose of estimating body mechanics needed for later analysis (moment of inertia, mass, height of center of mass, CoM) [22].

**Table 1:** Aim 1 CSMI test conditions and sensory weight comparisons

Condition	Vision	Support Surface	Visual Surround	Sensory Weight Comparisons
1	EC	PRS	--	*W*_*prop*_ vs. *W*_*vest*_
2	EO	PRS	Fixed	*W*_*prop*_ vs. *W*_*vis*_ *+ W*_*vest*_
3	EO	Fixed	PRS	*W*_*vis*_ vs. *W*_*prop*_ *+ W*_*vest*_
4	EO	PRS	PRS	*W*_*prop*_ *+ W*_*vis*_ vs. *W*_*vest*_

*EC* eyes closed, *EO* eyes open, *PRS* pseudorandom stimulus

#### Primary outcome measure

**Fig. 2:**
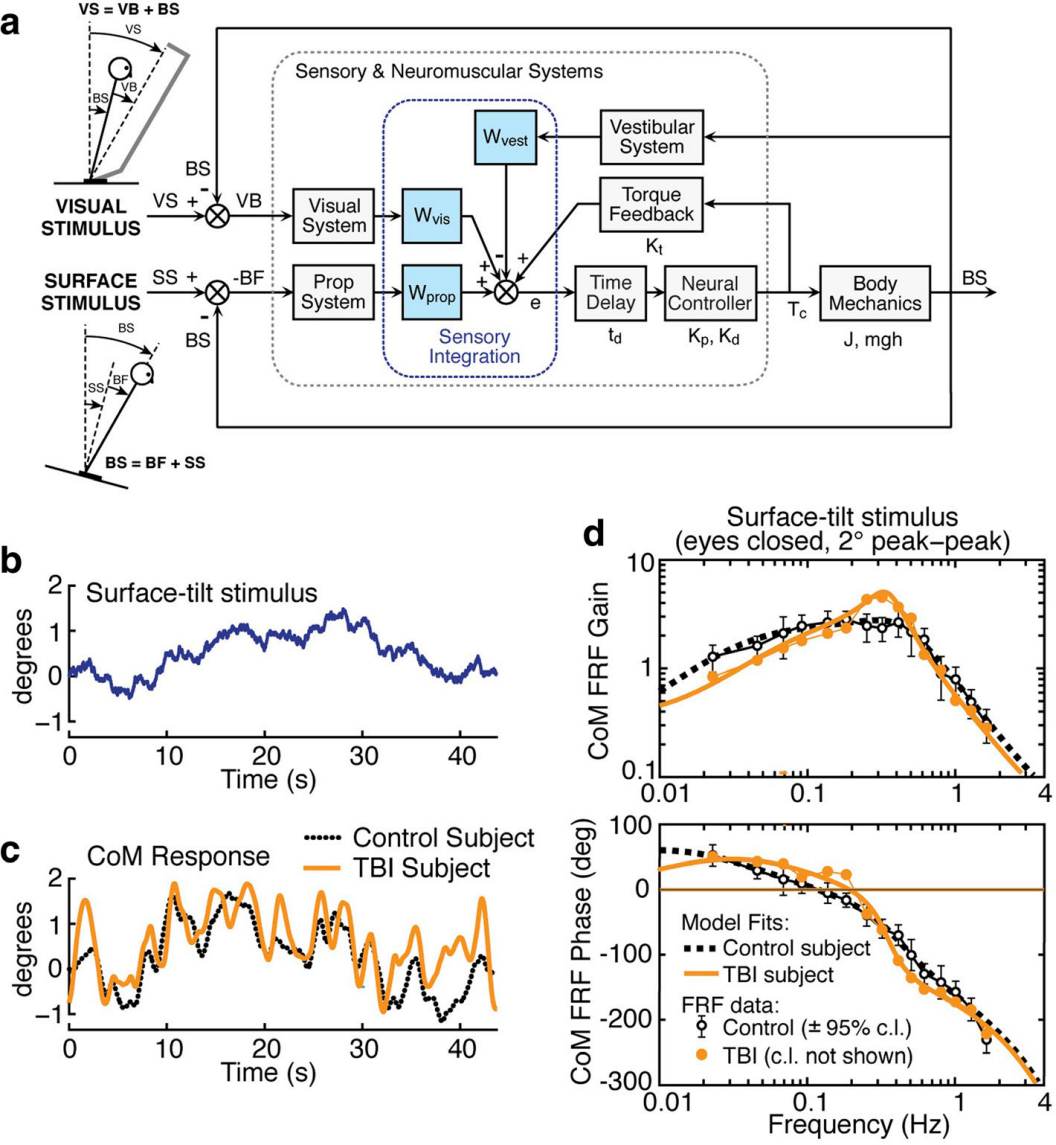
CSMI methods with example data and analysis. (**a**) A feedback control model forms the basis for identifying model parameters (Table 2) that account for experimentally evoked body-in-space (BS) sway, representing angular tilt of the body center-of-mass (CoM), evoked by support surface (SS) and/or visual surround (VS) rotations. (**b**) An example of one cycle of a pseudorandom surface-tilt stimulus that evoked the CoM body sway (averaged across 5 stimulus cycles) shown in (**c**) for a Control subject and TBI subject. (**d**) Frequency domain analysis of stimulus/response data in (**b**) and (**c**) yields frequency response functions (FRFs) expressed as gain (ratio of CoM response amplitude to stimulus amplitude) and phase (timing of response relative to stimulus) measures as a function of frequency components in the pseudorandom stimulus. Parameters of the model in (**a**) are calculated by a fit procedure that finds parameters that optimally account for the FRF gain and phase data

The primary outcome measures of central sensorimotor integration are the parameters derived from a model-based interpretation of CoM sway evoked by pseudorandom stimuli (Table 2). The procedure for calculating these parameters is to (1) calculate CoM angular displacement from experimental measures of hip and shoulder displacement on each test trial, (2) use Fourier methods to calculate a frequency response function, FRF (e.g. Fig. 2d) [46], and (3) adjust parameters of a model of the balance control system represented by the Fig. 2a block diagram using a constrained optimization algorithm (Matlab “fmincon” function; The Mathworks, Inc., Natick, MA) until the model-derived FRF optimally matches the experimental FRF (e.g. model-derived FRFs in Fig. 2d) [22, 47]. This analysis procedure will be applied to each of the 8 individual tests performed by each subject. The full set of model parameters are defined in Table 2.

**Table 2:** Balance control model parameters

*W*_*vis*_ *, W*_*prop*_ *, W*_*vest*_	Sensory weights of visual, proprioceptive, and vestibular contributions
*K*_*p*_ *, K*_*d*_	Neural controller parameters (sensory-to-motor transform)
*K*_*t,*_	Gain constant (torque feedback pathway)
*t*_*d*_	Net feedback time delay

The parameters include sensory weights that represent the relative contributions of proprioceptive, visual, and vestibular sensory information to balance control such that in each of the 4 test conditions the sum of weights equals 1. These weights are known to vary with test conditions that alter the availability (e.g. eyes open or closed) or reliability (e.g. varying stimulus amplitudes) of sensory information and are affected by sensory abnormalities (22, 24). For condition 1 tests (surface tilt stimulus with eyes closed) the model fit to the experimental FRF provides a measure of the proprioceptive contribution (Wprop). With eyes closed, the visual contribution (Wvis) is zero and therefore the vestibular contribution is Wvest = 1 – Wprop. Condition 2 tests also use a surface tilt stimulus but eyes are open with both visual and vestibular cues providing accurate orientation information. The model fit again provides an estimate of Wprop but now 1 – Wprop = Wvis + Wvest. Condition 3 tests present a visual tilt stimulus where the model fit provides a measure of Wvis and 1 – Wvis = Wprop + Wvest. Finally condition 4 tests simultaneously present the same surface and visual-tilt stimulus. In this condition the model fit provides a measure of Wprop + Wvis with Wvest = 1 – (Wprop + Wvis).

The neural controller parameters Kp and Kd (Table 2) represent the transformation from an internal sensory error signal (i.e. deviation from a desired body orientation) to a corrective ankle torque the moves the body toward a body orientation that reduces the sensory error. The neural controller parameters (Kp and Kd) are known to scale with subject anthropometrics [22] and, therefore, will be normalized prior to performing across subject comparisons. The balance model also assumes that a sense of effort contributes to the sensory error signal. This effort sense is represented by a torque feedback pathway with parameter Kt that is a scaling factor applied to the mathematical integration of the corrective ankle torque signal. This feedback pathway accounts for the low frequency characteristics of the FRFs that show gain declines and phase advances at frequencies below about 0.1 Hz (Fig. 2d). The time delay parameter td represents all delays in the system including sensory transduction, neural transmission, central processing, and muscle activation. The time delay parameter accounts for a substantial portion of the increasing FRF phase lag with increasing frequency.

#### Sample size

Power analysis for Study 1 was derived from a pilot dataset of 12 control subjects who participated in CSMI tests with the goal of estimating the sample size necessary for a normative reference group. This was defined as the number of control subjects necessary to see a coefficient of variation (CV) of less than 10%, a common threshold for measurement precision, in the CMSI outcome measures in order to develop a robust reference control group with a reasonable and acceptable amount of variability (49). Estimation was done with bootstrapping techniques using the stiffness control parameter (Kp) of the CMSI test of the pilot cohort as a sample distribution. For a considered sample size (ranging from 10 to 100 subjects), 1000 random samples with replacement were taken from the cohort’s Kp dataset to simulate a null distribution at that sample size and estimate the corresponding CV. Based on these permuted samples, the CV of the Kp parameter is expected to be less than 10% with 65 subjects, giving an adequately representative reference group. This same sample size of 65 will allow for a CV of ~6.5% in other CSMI measures such as visual sensory weighting. Therefore, a cohort of 65 mTBI subjects should be more than sufficient to properly identify any underlying classification differences arising from central sensorimotor integration abnormalities.

#### Secondary outcome measures

Secondary outcome measures of symptomology, neurocognition, static and dynamic balance, and central sensorimotor integration are provided in Table 3.

**Table 3:** List of all outcome measures by domain

Domain Tested	Test	Description	Clinical Outcomes	Instrumented Outcomes
Symptomology	Dizziness Handicap Inventory (DHI) [54]	Questionnaire about dizziness while performing various tasks	Total symptom score	-
	Post-Traumatic Stress Disorder (PTSD) Checklist [55]	List of problems and complaints people have in response to stressful life experiences	Total symptom score	-
	Pain Location Inventory (PLI) [56]	Questionnaire about presence and location of pain	Total symptom score	-
	SIQR Symptom Questionnaire [57]	Rates symptoms on a sliding scale	Total symptom score	-
	Sport Concussion Assessment Tool 3 (SCAT3) Symptom Questionnaire [58]	Rates 22 symptoms on a scale from 0 to 6	Total symptom score	-
	Becks Depression Inventory (BDI-II) [59]	Questions regarding depression and personal emotions	Total symptom score	-
	Short Form 36 (SF-36) [60]	36 questions about daily living and how symptoms have or have not changed over time	Total symptom score	-
	Neurobehavioral Symptom Inventory (NSI) [61]	Rates common symptoms associated with TBI	Total symptom score	-
Neurocognition	Automated Neuropsychological Assessment Metrics (ANAM) [62]	Computerized battery of neurocognitive tests	Individual component scores, Summary score	-
Reaction Time	Clinical Reaction Time Test [63]	Consists of dropping a rod attached to a weighted disk and measuring the distance the rod falls before being caught	Average distance the disk falls	-
Static Balance	Modified BESS [58]	20 s of stance with feet together, on one leg, and in tandem stance	Subjective error count	Measures of sway: RMS, jerk, total power, Mean distance, sway area
	Clinical Test of Sensory Organization and Balance (CTSIB) [64]	60 s of stance in 4 conditions: eyes open on firm ground, eyes closed on firm ground, eyes open on foam, eyes closed on foam	Number of conditions completed	Multi-scale complexity (entropy)
Dynamic Balance	Single-task gait	Walking at a comfortable pace down and back in a 16 m hallway for 16 times.	Gait speed, time to complete	Mediolateral trunk sway, Phase-coordination index, stride length, stride variability, nonlinear local dynamic stability
	Dual-task gait	Walking at a comfortable pace down and back in a 16 m hallway for 16 times while verbally responding to an auditory stroop cue	Change between single-task and dual-task gait speed	Change between single-task and dual-task instrumented outcomes
	Turning gait	Walking at a comfortable pace around a marked course 12 times	Average time to complete the course	Head-trunk-lumbar roll angular velocity, head-trunk-lumbar-foot yaw velocity, coordination of head-trunk-lumbar-foot reorientation
	Fast turning gait	Walking at a fast pace around a marked course 4 times	Change in time to complete course between comfortable and fast walking speeds	Change in turning gait instrumented measures between comfortable and fast walking speeds
Central Sensorimotor Integration	Sensory Organization Test (SOT) [65]	Calculates visual, proprioceptive, and vestibular ratio scores based on sway under varying sensory conditions	Visual, proprioceptive, and vestibular ratio scores	-
	Novel CSMI Test [22]^a^	Quantifies sway response to pseudorandom stimuli by calculating sensory weighting and neural controller parameters	-	Parameters defined in Table 2
Daily Activity	At-home activity monitoring^b^	Uses an inertial sensory worn daily to quantify the activity level	-	Activity level, number of steps per day, number of turns per day

^a^Primary outcome measure for Study 1 and Study 2

^b^Secondary outcome measure for Study 2 only

#### Static and dynamic balance testing

Subjects will be instrumented with body-worn inertial sensors during the performance of static and dynamic balance tests. Five synchronized wireless Opal inertial sensors (APDM, Inc., Portland, OR) will be affixed to the participants’ head, sternum, lumbar, left and right feet using elastic straps. Data will be collected at 128 Hz and transferred to a laptop for automatic generation of gait and balance measures by Mobility Lab software [48] and additional analysis of the raw time-series data. Specific tests and instrumented measures of static and dynamic balance are presented in Table 3.

#### Clinical testing for classification and covariates

To classify mTBI participants as with or without vestibular dysfunction, standard vestibular testing will be performed as described in Table 4. Clinical tests to examine potential covariates of sensory loss will also be included at the initial visit in Study 1.

**Table 4:** List of clinical sensory and vestibular tests for classification and covariates / comorbidities of primary and secondary outcomes

Domain Tested	Test	Description	Positive Result
Sensory	Proprioception test	Assess the ability of the participant to detect the position of the great toe or ankle as it is moved by a researcher / clinician	Unable to detect changes in position
	Visual Vertical Test	Requires participant to orient a line to vertical and quantifies the deviation from vertical	Falls outside control group mean ± 2 SD
Ocular Motor	Saccadic Latency	Assesses the ability to make a rapid eye movement, latency would be the time it takes to initiate a saccade (eye movement) after the target has moved	Falls outside control group mean ± 2 SD
	Gaze	Assesses the ability to maintain stable vision on a fixed target.	
	Smooth Pursuit	Assesses the ability to accurately track/follow a moving/sinusoidal target with the eyes.	
	Convergence	Assesses the ability to view a close target without double vision	
Vestibular	cVEMP	Neurophysiological test assessing the function of the saccule	Falls outside control group mean ± 2 SD
	oVEMP	Neurophysiological test assessing the function of the utricle	
	Caloric	Assesses function of the lateral semi-circular canals and superior branch of the vestibular nerves	
	Head Impulse	Assesses function of semi-circular canals and vestibular nerve branches, specifically the lateral canals/superior vestibular nerve	

#### Statistical analysis

To determine if objective/novel measures of balance will better distinguish people with mTBI from control subjects than clinical measures, we will use logistic regression classification models. Improvements in classification will be determined by calculating the area under the curve (AUC) of the receiver-operator characteristic and then comparing AUC for each traditional clinical measure with the novel objective measures. Significance will be assessed using bootstrapping to determine if the observed differences in AUC are greater than would be expected by chance at a level of 0.05.

To determine if a subset of people with mTBI will have abnormal CSMI test measures, even without peripheral vestibular or ocular motor deficits, we will use descriptive statistics to characterize the distribution of CSMI test measures and vestibular/ocular motor scores. We will define CSMI test measures and vestibular/ocular motor scores as abnormal if the scores are greater than 2 standard deviations from the mean in controls. We will then classify mTBI subjects into one of 4 CSMI-vestibular/ocular motor categories 1) abnormal CSMI –abnormal vestibular/ocular motor scores, 2) abnormal CSMI –normal vestibular/ocular motor scores, 3) normal CSMI –abnormal vestibular/ocular motor scores, 4) normal CSMI –normal vestibular/ocular motor scores.

To determine if the observed relationship between poorer static/dynamic balance performance and mTBI is regulated/mediated by central sensorimotor integration, we will use mediation analysis. Mediation analysis [49] will allow for the partitioning of the association between mTBI and chronic imbalance into two parts, the direct relationship of mTBI on balance impairment as well as the causally mediated effect of mTBI on central sensorimotor integration, which, in turn is having the actual effect on imbalance. These varying generative mechanisms of balance deficits will be assessed by utilizing linear models relating mTBI to CSMI test measures alongside models of mTBI and CSMI test measures jointly affecting imbalance using the apportionment methods described by Baron and Kenny [49].

### Study 2: Rehabilitation of balance deficit

#### Assessment procedures

**Fig. 3:**
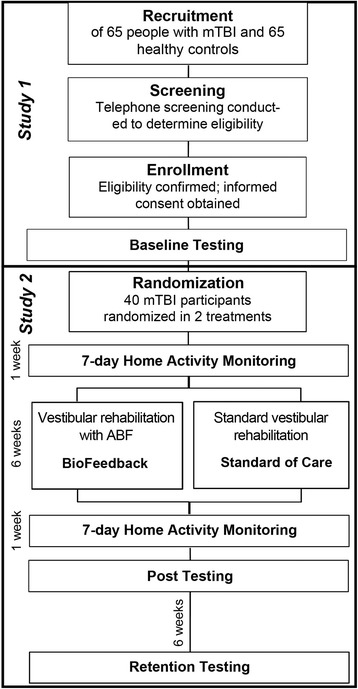
Flowchart illustrating the study design. Study 1 is a cross-sectional design. Study 2 recruits subjects from Study 1 for a randomized intervention

Subjects with mTBI who participate in Study 1 will be screened for Study 2. An investigator will verbally explain the consent form and time commitments involved with the rehabilitation, allow the person ample time to read through the consent form and then will acknowledge consent by signing the form. For Study 2, 40 mTBI participants will participate in a 6-week rehabilitation program randomized to either 1) a standard vestibular focused rehabilitation program or 2) the same standard vestibular focused rehabilitation program with the addition of audio biofeedback. Post-rehabilitation assessments will be conducted one week after the conclusion of the program. Retention will be assessed 6 weeks following the post-rehabilitation session. A flowchart illustrating the study design is depicted in Fig. 3.

#### Intervention

Participants will be randomized into either 1) a standard of care vestibular rehabilitation program or 2) a standard or care vestibular rehabilitation plus ABF training program. Rehabilitation will begin approximately 7-10 days after pretest assessment. Both groups will receive the rehabilitation that includes progressive (3 levels of difficulty) exercises that target gaze stabilization, vestibular stimulation, balance and proprioceptive retraining [50–52] (Table 5). Subjects randomized to the ABF training, will undergo the same vestibular rehabilitation program but will wear an ABF device while performing the rehabilitation exercises. All researchers performing post-rehabilitation testing will remain blinded to the randomized group assignment throughout the duration of the study.

**Table 5:** Rehabilitation protocol

Exercise	Vision	Surface	Time	Progression	ABF
Static Balance	Eyes open	Firm	30 s per condition	10 double stance conditions including tossing a ball, head rotations left-right, head rotations up-down, head rotations with smooth pursuit, with gaze stabilization, and with saccades	Pitch modulated feedback for AP sway, direction modulated for ML sway
	Eyes closed	Firm	30 s per condition	3 double stance conditions including head rotations left-right, head rotations up-down	
	Eyes open	Foam	30 s per condition	4 double stance conditions including tossing a ball, head rotations left-right, head rotations up-down	
	Eyes closed	Foam	30 s per condition	3 double stance conditions including head rotations left-right, head rotations up-down	
Dynamic Balance (Tandem Gait)	Eyes open	Firm	30 s per condition	4 tandem gait conditions including tossing a ball, head rotations left-right, head rotations up-down	Direction modulated for ML sway
	Eyes closed	Firm	30 s per condition	1 tandem gait condition	
	Eyes open	Foam	30 s per condition	4 tandem gait conditions including tossing a ball, head rotations left-right, head rotations up-down	
	Eyes closed	Foam	30 s per condition	1 tandem gait condition	
Dynamic Balance (Bending down)	Eyes open	Firm	30 s per condition	3 different heights	Direction modulated for ML sway
	Eyes closed	Firm	30 s per condition	3 different heights	
Dynamic Balance (Squatting)	Eyes open	Firm	30 s per condition	4 squats including sit-to-stand, lunge, lunge onto unstable surface, and lunge + twist	Direction modulated for ML sway
	Eyes closed	Firm	30 s per condition	4 squats including sit-to-stand, lunge, lunge onto unstable surface, and lunge + twist	
	Eyes open	Foam	30 s per condition	4 squats including sit-to-stand, lunge, lunge onto unstable surface, and lunge + twist	
	Eyes closed	Foam	30 s per condition	4 squats including sit-to-stand, lunge, lunge onto unstable surface, and lunge + twist	

#### Auditory biofeedback

ABF will be supplied by a lumbar-mounted smartphone (mHealth Technologies s.r.l., Bologna, Italy). The ABF system detects AP and mediolateral (ML) linear accelerations near the body’s CoM. The participant wears headphones and the ML inclination with respect to gravity is encoded as a sound in either the right or left ear while the AP tilt is encoded as changes in pitch as the person leans forward or backward. When the body is in perfect equilibrium, the system is quiet. A calibration test is used to determine optimal balance parameters such that the auditory feedback increases only when the person exceeds their baseline sway by 1° or the tilt of the trunk during gait exceeds an established threshold [42, 53]. Both AP and ML feedback will be supplied to the participant during static balance exercises. During dynamic balance exercises, only ML feedback will be provided. During balance exercises, participants will be instructed to utilize the auditory sounds provided by the ABF system to maintain balance and keep the system quiet (i.e., minimal deviations from the stable calibration).

#### Primary and secondary outcome measures

The primary outcome measure is the change between pre-rehabilitation, post-rehabilitation, and 6-week follow-up testing of the model-based parameters of the novel CSMI test (Table 2). Secondary outcome measures are the changes between pre-rehabilitation, post-rehabilitation, and 6-week follow up testing of the clinical and objective balance measures described in Study 1 and in Table 3. Additional secondary outcome measures from the at-home inertial sensors will include activity rate and average turning speed and number of turns.

#### Sample size

We powered the intervention study to detect improvement in the CSMI test deficit we found in our pilot subjects. Conservatively, 20 subjects will be recruited for both the experimental treatment and standard of care groups to provide results that are capable of powering a more in-depth future clinical trial investigating rehabilitation for balance deficits in mTBI patients. The CSMI test deficits observed in 3 mTBI pilot subjects and 12 healthy controls were used to power the study. Specifically, the CSMI parameter Kp under open eye surface tilting (t = -2.02, *p* = 0.065) and visual sensory weight Wvis under fixed surface visual tilting (t = 2.07, *p* = 0.060) were used to calculate sample size based on independent sample t-tests. Rehabilitation-based differences within the TBI cohort are not expected to be as extensive as those identified between mTBI subjects and controls. However, an effect size at 50% of the magnitude of the difference observed between mTBI and controls in the preliminary data (Coehn’s d = 0.748) would still yield 72% power with α = 0.10 in a repeated measures framework in as little as 10 subjects per group. Conservatively, we will recruit 20 subjects each for the experimental treatment and standard of care groups to provide results that are capable of powering a more in-depth future study investigating rehabilitation for balance deficits in mTBI patients.

#### Statistical analysis

To determine if ABF intervention improves central sensorimotor integration, we will build linear regression models to simultaneously evaluate the change in CSMI measures reflecting central sensorimotor integration and the effect of ABF compared to standard of care. Our outcome will be the change in pre- versus post-rehabilitation program CSMI measures; using the change (difference) in CSMI measures will control for the correlation of the repeated pre- and post- measures within an individual as well as maximizing the number of covariates we can potentially add into the regression models. Covariates of interest are vestibular/ocular motor scores, PTSD, Veteran versus civilian status, age, and gender. In addition, we will determine whether rehabilitation improved the static and dynamic balance metrics that were measured at the pre- and post-rehabilitation testing visits.

To determine if people with CSMI impairment show sustained improvement in static and dynamic balance after rehabilitation, even after ABF is discontinued, we will use linear mixed effects regression modeling which will include a third time point of interest (6 weeks after the rehabilitation intervention has stopped). Time will be treated as a categorical variable in order to evaluate changes in CSMI scores and balance during specific time periods, in this case, between the cessation of the intervention and the 6 weeks post-intervention follow-up. We will explicitly test the significance of a group by time interaction to determine if participants in the ABF rehabilitation group showed sustained improvements in outcomes beyond that experienced by participants with the standard of care. If applicable, we will then stratify the analysis by those with abnormal/normal measures of vestibular/ocular motor function to assess the relationship between vestibular/ocular motor function deficits and sustained improvement in CSMI score and balance.

## Discussion

The goal of this study is to determine whether persistent balance and gait deficits following mTBI are attributable to improper central sensorimotor integration and to examine the potential for augmented rehabilitation to improve daily activity levels. In particular, this study is unique in its investigation of central sensorimotor integration in people with persistent balance complaints following mTBI in that confounding covariates such as vestibular disruption, neurocognition, and physical activity will be objectively quantified. Additionally, if we find that the quantitative balance, gait, or novel CSMI measures have a significantly greater AUC compared to the clinical measures (BESS, gait speed, SOT) at detecting patients with chronic mTBI, this will indicate the necessity for objective quantification of gait and balance deficits for mTBI assessment.

If a portion of subjects with chronic balance problems after mTBI have abnormal CSMI test measures but normal clinical vestibular testing results, this suggests that sensory integration processes in the brain can be impaired following mTBI, even without peripheral sensory deficits, and that abnormal central sensorimotor integration plays a key role in persistent abnormal balance in subjects with normal vestibular function. If a portion of mTBI subjects have abnormal CSMI test measures and abnormal clinical vestibular function, the key mediator for balance problems is less clear and may be due to the peripheral vestibular dysfunction, abnormal central sensorimotor integration, or inadequate adaptation of central sensorimotor integration to account for abnormal sensory information. If we find that abnormal central sensorimotor integration mediates abnormal static or dynamic balance, rehabilitation will need to be focused on improving central sensorimotor integration to improve balance. If we find that CSMI scores improve with rehabilitation, we will conclude that central sensorimotor integration strategies are flexible and can improve with training. Further, if we find that central sensorimotor integration and gait and balance jointly improve with rehabilitation, we will have further evidence that central sensorimotor integration plays a key role in the mediation of gait and balance following mTBI. If we find a greater improvement in the ABF group, we will conclude that ABF may facilitate improved rehabilitation. Finally, if we find that people with central sensorimotor integration impairment maintain improvements after stopping the use of ABF device, we will conclude that a recalibration of sensory integration and/or sensorimotor transformation for balance has occurred, rather than sensory substitution that requires active ABF. If we find that sensory strategies can change with practice and biofeedback, we will conclude that this targeted approach to balance rehabilitation should be explored further in a larger, randomized trial. It is possible that different subclasses based on CSMI measures respond differently to rehabilitation or ABF-augmented rehabilitation. In such a case, the results of this study will provide valuable information for powering an in-depth future study carefully examining the relationship between CSMI subclasses and the response to rehabilitation with and without ABF. The results of this study will further our understanding of the mechanisms underlying posture and gait dysfunction in individuals with chronic mTBI and inform future studies about the efficacy of ABF augmented rehabilitation programs.

## Abbreviations

ABF: Auditory biofeedback
ANAM: Automated neuropsychological assessment metrics
AP: Anteroposterior
AUC: Area under the receiver-operator characteristic curve
BDI-II: Becks depression inventory
BESS: Balance error scoring system
CoM: Center of mass
CSMI test: Central sensorimotor integration test
CTSIB: Clinical test of sensory organization and balance
CV: Coefficient of variation
DHI: Dizziness handicap inventory
FRF: Frequency response function
LOC: Loss of consciousness
ML: Mediolateral
mTBI: Mild traumatic brain injury
NSI: Neurobehavioral symptom inventory
PLI: Pain location inventory
PTSD: Post-Traumatic stress disorder
RMS: Root-mean-square
SCAT3: Sport concussion assessment Tool 3
SF-36: Short form 36
SOT: Sensory organization Test
TBI: Traumatic brain injury.
